# Examining Land Use Changes to Evaluate the Effects of Land Management in a Complex, Dynamic Landscape

**DOI:** 10.1007/s00267-020-01316-2

**Published:** 2020-06-22

**Authors:** Amanda K. Martin, Karen V. Root

**Affiliations:** Department of Biological Sciences, Bowling Green State University, Bowling Green, OH 43403 USA

**Keywords:** Conservation, Land cover change, Oak Openings Region, Urbanization, Vegetation classification

## Abstract

Anthropogenic alterations to landscapes have increased as the human population continues to rise, leading to detrimental changes in natural habitats. Ecological restoration assists in recovery by altering habitats to improve conditions and foster biodiversity. We examined land cover changes over time within a complex, dynamic region in the Midwest to assess the long-term effects of conservation. We used Landsat 8 bands for a 15-class land cover map of Oak Openings Region using supervised classification. We validated our map and achieved an overall accuracy of 71.2% from correctly classified points out of total visited points. Change over 10 years, from 2006 to 2016, was explored by comparing class statistics from FRAGSTATS between our map and original land cover map. We found that natural land, i.e., forest and early successional, covered 33%, with 10% permanently protected, while human-modified land, i.e., agricultural and developed, covered 67% of the region. Over 10 years, natural classes increased, and cultural classes decreased by 5.8%. There were decreases for the three forest communities and increases for the two early successional communities. These changes are likely the result of natural recovery and disturbance, and conservation efforts by the Green Ribbon Initiative. Changes in habitat also came with distribution changes, e.g., increased fragmentation for some classes, which was readily visible. Our useful method measured functionality by emphasizing changes in composition and configuration. Our approach provides a tool for assessing cumulative regional-scale effects from site-level management and conservation. This large-scale view for conservation is needed to effectively mitigate future changes.

## Introduction

Growing anthropogenic pressures continue to intensify and affect natural and semi-natural ecosystems. As these pressures increase, there is a greater drive to quantify land-use land cover (LULC) changes. Since LULC is one of the most important variables that affects global ecosystems (e.g., Lovell and Johnston 2009; Srivastava et al. 2012; Cordell et al. 2016). There are many alterations in modern landscape structure, many of which are expected to be fairly permanent with continued urbanization. However, these changes without some level of intervention, such as land acquisition and then restoration, can lead to the loss of critical ecosystem functions (O’Farrell and Anderson 2010). Such as local extinctions of plants and their associated animals, as seen in converting temperate grasslands into croplands or forests into grasslands (Sala et al. 2000). These abrupt environmental changes occur at different scales (e.g., local, regional, and global) and have been increasing over time as a result of both natural and anthropogenic processes. However, many of these changes are especially driven by urbanization which continually fragment and reduce natural habitats. Urbanization and human activities have expedited ecosystem(s) suffering from degradation, damage, and/or destruction, which has led to a larger focus on ecological restoration (Sala et al. 2000; Matzek et al. 2017). Many local organizations that focus on providing stronger support for restoration and conservation efforts require different tools that foster an assessment of change from the past, current, and future potential, especially for these dynamic landscapes. In particular, land managers need a way to assess the effects of their efforts across scales (temporal and spatial), or a way to put local scale management (i.e., individual parcels) into larger-scale conservation targets across landscapes (e.g., watersheds).

In a region where there are remnant natural habitat patches in a matrix of human-modified habitat types, e.g., agriculture, residential, there is a need to actively manage threats such as invasive species, fire suppression, water diversion, and human disturbance. An example of one of these dynamic yet diverse landscapes is Oak Openings Region within southeastern Michigan and northwestern Ohio, USA, described in more detail below. Large conservation efforts have focused on examining the changes within this region (e.g., Schetter et al. 2013; Abella et al. 2017), and there have been significant alterations as a result of anthropogenic activities (e.g., development, restoration), natural disturbance (e.g., tornado) and invasive species (e.g., emerald ash borer, *Agrillus planipennis*, Fairmaire). Traditional approaches towards ecological restoration include the community and ecosystem approach. For an example, targeted management practices focus on a few select species, such as the listed Karner Blue Butterfly (*Lycaeides milissa samuelis*, Nabokov), or on a particular ecosystem, e.g., oak savanna, and manage for those targets using prescribed fire, mowing, etc. (Pickens and Root 2008; Pickens and Root 2009).

In Oak Openings Region there are several globally imperiled ecosystems, including oak savanna, which are the focus of conservation efforts (Abella et al. 2007; Schetter et al. 2013). The Green Ribbon Initiative (GRI) is a local conservation group that brought together multiple agencies in a partnership to protect the Oak Openings Region’s natural beauty and biological diversity (https://oakopenings.org). The GRI mission is to enhance and restore critical natural areas and has focused on the conservation of five target ecosystems: upland savanna/prairie, wet prairie, upland deciduous forest, floodplain forest, and flatwoods/swamp forest (Gardner 2016). These ecosystems are especially vulnerable because of threats from invasive species such as emerald ash borer for forest (Kashian and Witter 2011; Herms and McCullough 2014), woody encroachment (Eldridge et al. 2011) for temperate grassland, altered fire regimes for oak savanna (Peterson and Reich 2001), and channel drainage for wet prairie (Wijayarathne 2015). Ecosystem-based management, such as targeting the five major communities in Oak Openings Region (Supplement Figure 1), aims to better protect dynamic and functional habitats that support a diverse array of species. In addition, land managers can incorporate target species associated with specific ecosystems in order to facilitate conservation. In this region, active management is used to combat current threats, as well as continued land acquisition, and restoration to help restore functionality.

Spatial tools are invaluable in guiding the assessment of restoration efforts, evaluating management options, identifying conservation priorities, and quantifying threats across various scales (Cordell et al. 2016). The landscape ecology approach emphasizes structure, e.g., spatial heterogeneity, function, connectivity, and can potentially help identify the aggregate effects of site scale management across a region (Bell et al. 1997; Ehrenfeld and Toth 1997; Lovell and Johnston 2009; O’Farrell and Anderson 2010; Cordell et al. 2016). LULC changes have been monitored by traditional field inventories and surveys, but satellite remote sensing provides an accessible and efficient method of acquiring information on temporal and spatial trends (Yuan et al. 2005; Lovell and Johnston 2009). In terms of cost, time, and area coverage, quantifying these changes from Landsat data can be more effective than field surveys (El Baroudy and Moghanm 2014) and potentially provide a broader perspective on functionality (Lovell and Johnston 2009).

In addition to measuring LULC changes, integrating landscape-pattern metrics from FRAGSTATS with Geographic Information System (GIS) mapping can enhance landscape analysis by quantifying structural changes within ecosystems at various spatial scales (Lovell and Johnston 2009). Changes in both composition and configuration are invaluable for restoration efforts especially for heterogeneous landscapes that vary in the types and proportions of different land covers and how they are arranged on the landscape. The landscape is defined by this heterogeneity and has varying impacts on biodiversity (Fahrig 2003; Fahrig et al. 2011; Tscharntke et al. 2012). These are examined through the lens of area, density, edge, shape, proximity, interspersion, connectivity and diversity metrics, which include parameters such as number of patches, perimeter-area ratio, patch richness, etc. (Torbick et al. 2006; Driezen et al. 2007; Schetter et al. 2013; Herse et al. 2018). These metrics, specifically landscape and class-level, are reliable for assessing natural and/or anthropogenic LULC changes that disrupt abiotic or biotic landscape structure including management activities (Gottgens et al. 1998; Lopez et al. 2002; Houlahan and Findlay 2004; Johnston and Rejmánková 2005). In addition, class-level spatial patterns are important for species conservation (Murphy and Noon 1992; Villard et al. 1999) as a result of their high correlations with various ecological processes. For an example, patch cohesion captures a biologically relevant aspect of landscape structure (i.e., habitat connectivity) by evaluating multiple landscape structural properties on a continuous scale. In addition, it can be used to estimate partial habitat suitability values for multiple species dependent on similar habitat, but with varying abilities and spatial scale (Wilson 2007).

We examined landscape changes in terms of composition and configuration over a 10-year period, from 2006 to 2016, for the highly heterogeneous Oak Openings Region in Northwest Ohio and Southeast Michigan. We wanted to highlight where and how much land cover changed to better aid local conservation efforts towards prioritization, land acquisition, restoration, and management. In addition, we established a generalizable model for similar restoration efforts for other dynamic landscapes. We anticipated that we should see improvement in the extent of the five focal ecosystems within protected areas as a result of targeted management activities. We also expected that human-modified land cover types would increase with expanding urbanization outside of protected lands. Our objectives were to: (1) create an updated land cover map; (2) compare changes in land cover over time; and (3) provide a tool to evaluate conservation efforts including acquisition, restoration, and land management. We utilized the original Northwest Ohio land cover map based on 2006 data (Schetter and Root 2011) to compare the relative changes over time with the updated Northwest Ohio land cover map based on 2016 data; however, we emphasized the five focal communities targeted for restoration and conservation. We tested the following predictions as a result of the restoration efforts of local land managers. We first predicted that natural areas (especially for the five major communities of concern) within protected lands will have increased over time because of restoration and land acquisition efforts. We then predicted that number of patches and edge density will decrease, while average patch area and the largest patch size will increase over time as a result of efforts towards increasing connectivity between protected areas. Our study is unique through the incorporation of both composition and configuration metrics, i.e., total area, connectivity, to measure changes in functionality over time.

## Methods

### Study Area

The full extent of Oak Openings Region, mapped area of 194,000 ha, ranges from Northwest Ohio including Henry, Fulton and Lucas counties to Southeast Michigan including Monroe, Wayne, Washtenaw, Wayne, and Oakland counties (41° 27′ to 42° 10′N; 83° 51′ to 83° 32′W). Although this landscape is highly fragmented, it contains five globally rare communities (Brewer and Vankat 2004) that have great conservation value as a result of the amount of rare species and unique habitats. For historic and detailed description of land cover classes refer to Schetter and Root (2011). It is a highly heterogeneous landscape that lies within anthropogenic lands (e.g., agriculture, residential, urban), yet it remains a local biodiversity hotspot for state endangered, threatened, potentially threatened species (e.g., Sweet-fern (*Comptonia peregrine*, Coult), Showy Lady’s-slipper (*Cypripedium reginae*, Walter), Grass-pink orchid (*Calopogon tuberosus*, Britton, Sterns, and Poggenb), Blunt-leaved milkweed (*Asclepias amplexicaulis*, Sm.)). Several endangered or threatened species, such as the Karner Blue Butterfly and northern long-eared bat (*Myotis septentrionalis*, Trouessart), depend on the rare natural communities found in this region. Despite the small size (Ohio portion 47,800 ha, Michigan portion 146,200 ha), Oak Openings Region contains nearly a third of Ohio’s rare plants and animals (Schetter et al. 2013).

### Landsat 8 Image Selection

We acquired three multi-season images from the United States Geological Survey (USGS) Earth Explorer (USGS 2017) for the early, mid, and late growing season. The three images were from Landsat 8 (Tier 1) on 16 April 2016, 19 June 2016, and 9 October 2016 for Path 20, Row 31, which contained our entire study area. Multi-seasonal imagery was especially useful for examining dynamic vegetation patch patterns and improved classification for forest cover (Gudex-Cross et al. 2017; Clark et al. 2018; Higginbottom et al. 2018), grassland (Poulin et al. 2010; Wang et al. 2010; Dusseux et al. 2014), land use/land cover changes (Löw et al. 2015; Nitze et al. 2015; Zhao et al. 2016) and more (Liu et al. 2019). We required each image: (1) occurred within a narrow 6-month timeframe, (2) featured 0% cloud cover within our study area, and (3) contained Level-1 Precision Terrain (L1T) images. We downloaded all images in GeoTif image format and projected to Universal Transverse Mercator coordinates (UTM Datum WGS84). The Landsat 8 images were multispectral; however, the 1-Coastal Aerosol, 8-Panchromatic, and 9-Cirrus bands were excluded in our final classification to more accurately compare to Schetter and Root (2011) classification, hereafter referred to as the original map. The original map used three multi-seasonal Landsat-5 TM scenes acquired in 2006.

### Training Site Selection

We used the original Ohio training sites from Schetter and Root (2011) 2006 land cover classification and included novel training sites, these are sites that were not used in the 2006 land cover classification, within Ohio and Michigan collected by The Nature Conservancy. The original Ohio training sites were revisited to confirm land cover type after the 10-year period. We delineated training sites with enough training pixels for a supervised classification within ArcGIS 10.2 (Environmental Systems Research Institute, Redlands, California) across Oak Openings Region. We used 27 bands (9 per image), each with 30 m pixel resolution that required a minimum of 28 pixels per land cover class (*n* + 1 pixels required per class, where n is the number of used spectral bands). We used 132 training sites for 14 classes (average of 9 training sites per class).

### Supervised Image Classification

We performed a supervised classification for 14-land cover types that matched the original 2006 land cover classification using maximum likelihood classification model in ArcGIS 10.2. Cropland was difficult to classify because of seasonal changes in planted crops’ type and phenology. Therefore, we applied a cropland “mask” (USDA 2016) to the 14-land cover classification and produced the final image (as in Schetter and Root 2011). After we applied the mask, we clipped the 15-class image for Oak Openings Region (Gardner 2016) and then clipped the 15-class image to the historic extent of Oak Openings Region in Northwest Ohio for comparison to the original map (Brewer and Vankat 2004). The 15 landcover classes are listed in Table 1 with photographs for several major land cover classes (Supplementary Fig. 2). These land covers are distinguished by the class descriptions defined by Schetter and Root (2011). Briefly defined for some of the major land covers: swamp forest contains closed-canopy deciduous swamps/flatwoods that are semi-permanent to seasonal, whereas floodplain forests have closed to open canopy deciduous forest that are poor to moderately well drained. Wet shrublands have a well-developed herbaceous layer that is semi-permanent to seasonal on soils that are poorly drained. Upland savannas contain open canopy oak stands with well-drained soils and a developed herbaceous and shrub layer, dominated by warm-season grasses and forbs, whereas trees are nearly or completely absent within upland prairies and shrub layers are generally sparse or absent on these mesic to dry areas with warm-season grasses. Finally, wet prairies have nearly or entirely absent trees and shrubs and are semi-permanent to seasonal on poorly drained soils.

**Table 1:** Oak Openings land cover classification system for the 15-class, 7-class, and 5 target ecosystems

15 land cover classes	7 land cover classes	5 target ecosystems
Swamp forest (SF)	Forest & woodland	Swamp forest
Floodplain Forest (FF)		Floodplain forest
Upland Deciduous forest (UD)		Deciduous forest
Upland Coniferous forest (UC)		
Upland Savanna (US)	Savanna	Savanna/Prairie
Wet Shrubland (WS)	Shrubland	
Wet Prairies (WP)	Prairie & Meadow	Wet Prairie
Upland Prairie (UP)		Savanna/Prairie
Sand Barren (SB)		
Eurasian Meadow (EM)		
Water (PP)	Water	
Dense Urban (DU)	Built-up	
Residential/Mixed (RM)		
Turf/Pasture (TP)	Vacant	
Cropland (CR)		

### Accuracy Assessment

We assessed the accuracy of the classification with a combination of field surveys and orthophotos. Ground truth points for field surveys were created in ArcGIS 10.2 and were at least 150 m apart within protected lands to ensure a reasonable sample distribution. Points were selected within areas that were readily accessible for which we visited 270 points. Travel time was minimized by evaluating four adjacent neighboring points from the selected point. We visually inspected ground truth points within major communities of concern (e.g., upland savanna, upland prairie, wet prairie, upland deciduous forest, floodplain forest, and swamp forest), and identified if the land cover class was accurately designated by the supervised classification. The point was considered correctly classified if within 5 m of the area at the point matched the updated map classification and was marked as incorrect if it did not match. In addition, we verified the classification using high resolution (0.3 m) color orthophotos acquired in 2017 (USGS 2017). We used orthophotos for distinguishable, identifiable land cover classes (e.g., cropland, dense urban, residential/mixed, water, and upland coniferous forest) across the study region. We followed the same methodology as the field surveys by evaluating the four adjacent neighboring points for a total of 230 points. We evaluated overall accuracy for all 15-land cover types and 7-level land cover (class descriptions in Schetter and Root 2011), see Table 1 for the list of each set. We compiled the assessment results in an error matrix.

### Regional Assessment

We assessed land cover changes in Oak Openings Region for a 10-year period by comparing the updated map to the original map for three groups: a 15-level classification, a 7-level classification (defined in Schetter and Root 2011) and for the five major communities of concern, see Table 1 for the list for each set. The 15-land cover classes were combined to create the seven-level classes, which included forest and woodland (i.e., swamp forest, floodplain forest, upland deciduous forest, upland coniferous forest), savanna (i.e., upland savanna), shrubland (i.e., wet shrubland), prairie and meadow (i.e., wet prairie, upland prairie, sand barren, Eurasian meadow), water (i.e., perennial pond), built-up (i.e., dense urban, residential/mixed), and vacant (i.e., turf/pasture, cropland), Table 1. In addition, we evaluated changes among protected and unprotected lands using a protected lands shapefile layer (Northwestern Ohio Park Inventory). Using FRAGSTATS 4.2.1 (McGarigal and Marks 1995) we examined landscape-pattern metrics by compiling per-class data on the total area, average class area, number of patches, and the largest patch index. We examined in FRAGSTATS 4.2.1 estimates of connectivity (i.e., patch cohesion index), dispersion (i.e., clumpy) and heterogeneity (i.e., patch richness) using a moving window neighborhood analysis with a 120 m by 120 m window. Landscape-pattern metrics were evaluated first for the seven-level class model and then for the five major communities of concern.

### Restoration within Oak Openings Preserve

We examined restoration efforts through land cover change within Oak Openings Preserve over a 10-year period. We assumed that most of the changes that occurred during this period were linked to management actions, as this park is heavily managed. Targeted efforts have been removing and converting upland coniferous forest to early successional land cover among other interventions. We assessed these changes by compiling per-class data on the total area and number of patches delineated in FRAGSTATS 4.2.1.

## Results

### Map Characteristics

#### 15-class land cover

**Fig. 1:**
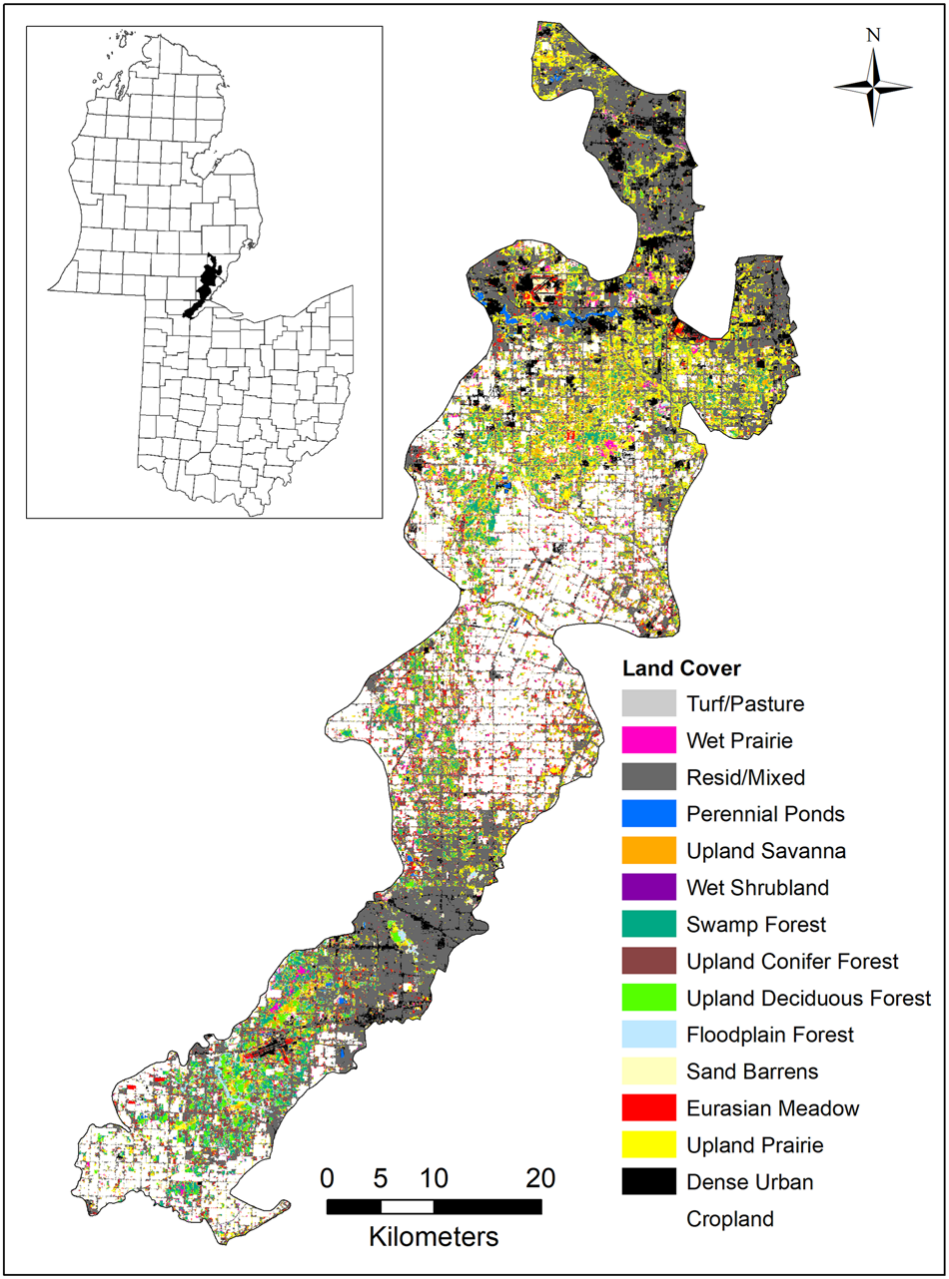
Map of the 15-class updated land cover map of Oak Openings Region in Northwest Ohio and Southeast Michigan

The final updated 15-class land cover map was classified for the full extent of Oak Openings Region, which includes Northwest Ohio and Southeast Michigan (Fig. 1). For all other analyses, we only used the portion contained in Northwest Ohio for comparison of changes over time to Schetter and Root (2011), which did not include Michigan. We found that natural/seminatural land cover classes in the updated map increased to 33% of the region, while cultural land cover classes decreased to 67% of the total area. We found that the total area (i.e., eight classes), average patch area (i.e., 11 classes), the number of patches (i.e., seven classes), and the largest patch index (i.e., 10 classes) increased over time (Table 2). Upland prairie had the greatest increase in the total area of 2502 ha and increased by 4260 patches, while turf/pasture had the greatest decrease in the total area of 3102 ha and decreased by 7115 patches. Residential/mixed had the greatest increase in the average patch area of 2.6 ha and increased by 5% in the largest patch index, while cropland had the greatest decrease in the average patch area of 31.5 ha and decreased by 0.1% in the largest patch index.

**Table 2:** Summary of land cover results using Total Area, Average (AVG) Patch Area, Number (#) of Patches, and the Largest Patch Index (LPI) for the seven-level class system and five major communities of concern for the original (Schetter and Root 2011) and updated map within Oak Openings Region in Northwest Ohio

	Total area (ha)		AVG area (ha)		# Patches		LPI (%)	
Class	2006	2016	2006	2016	2006	2016	2006	2016
Natural/Seminatural								
Forest and Woodland	9734	7021	3.7	*4.3*	2634	1617	3.43	2.7
Swamp Forest	1496	*3205*	0.4	*1.4*	3761	2235	0.09	*0.2*
Floodplain Forest	4259	1503	0.7	0.5	6189	3129	0.41	*0.4*
Upland Deciduous Forest	3073	1914	1.1	*1.2*	2937	1659	0.16	0.1
Upland Coniferous Forest	907	400	0.8	*1.2*	1133	330	0.2	0.1
Savanna (Upland Savanna)	370	*959*	0.2	*0.4*	1664	*2408*	0.05	*0.1*
Shrubland (Wet Shrubland)	193	5	0.3	*0.4*	732	14	0.05	0.0
Prairie and Meadow	2438	*7679*	0.5	*1.0*	5048	*7380*	0.14	*0.5*
Wet Prairie	40	*695*	0.4	0.3	104	*2400*	0.02	*0.1*
Upland Prairie	610	*3112*	0.2	*0.4*	2819	*7079*	0.04	*0.1*
Sand Barren	359	*2071*	0.2	*0.4*	1946	*5161*	0.01	*0.1*
Eurasians Meadow	1429	*1802*	0.4	*0.4*	3884	*4622*	0.09	0.1
Water (Perennial Pond)	253	236	0.9	*1.3*	290	186	0.05	*0.1*
Cultural								
Built-up	18749	*19669*	4.2	*7.3*	4445	2699	28.1	*33.2*
Dense Urban	1833	*1908*	1.1	0.9	1688	*2057*	0.33	*0.4*
Residential/Mixed	16915	*17761*	3.5	*6.1*	4812	2905	24.3	*29.3*
Vacant	16042	12189	3.3	*16.9*	4801	720	22	17.1
Turf/Pasture	3141	39	0.4	*0.6*	7183	68	0.1	0.0
Cropland	12901	12150	50.0	18.5	258	*657*	16.4	*17.1*

Italic numbers represent increases over time

#### Seven-class land cover

When we examined changes across our seven-level classes, we found that forest and woodland lost 5.4% of habitat, whereas prairie and meadow gained 10.9% of habitat. We additionally found that savanna and built-up increased, respectively, by 1.2 and 1.8%, whereas vacant and shrubland decreased, respectively, by 7.6 and 0.4% (Table 2). In terms of structure, we found that vacant, forest and woodland, shrubland, and water decreased in total area over time, while prairie and meadow had the greatest increase followed by built-up and savanna. We found that vacant, built-up and forest and woodland lost more than 1000 patches, while prairie and meadow increased by 2332 patches (Table 2). Over the 10-year period, the average patch area increased for each of the seven-level classes.

#### Five major communities of concern

**Fig. 2:**
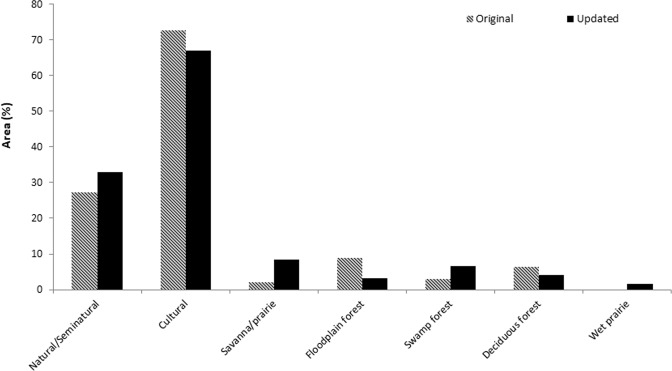
Percentage of area for all natural/seminatural, all cultural land covers and for each of the five major communities of concern for the original (pattern) and updated (no pattern) land cover map in Oak Openings Region

**Fig. 3:**
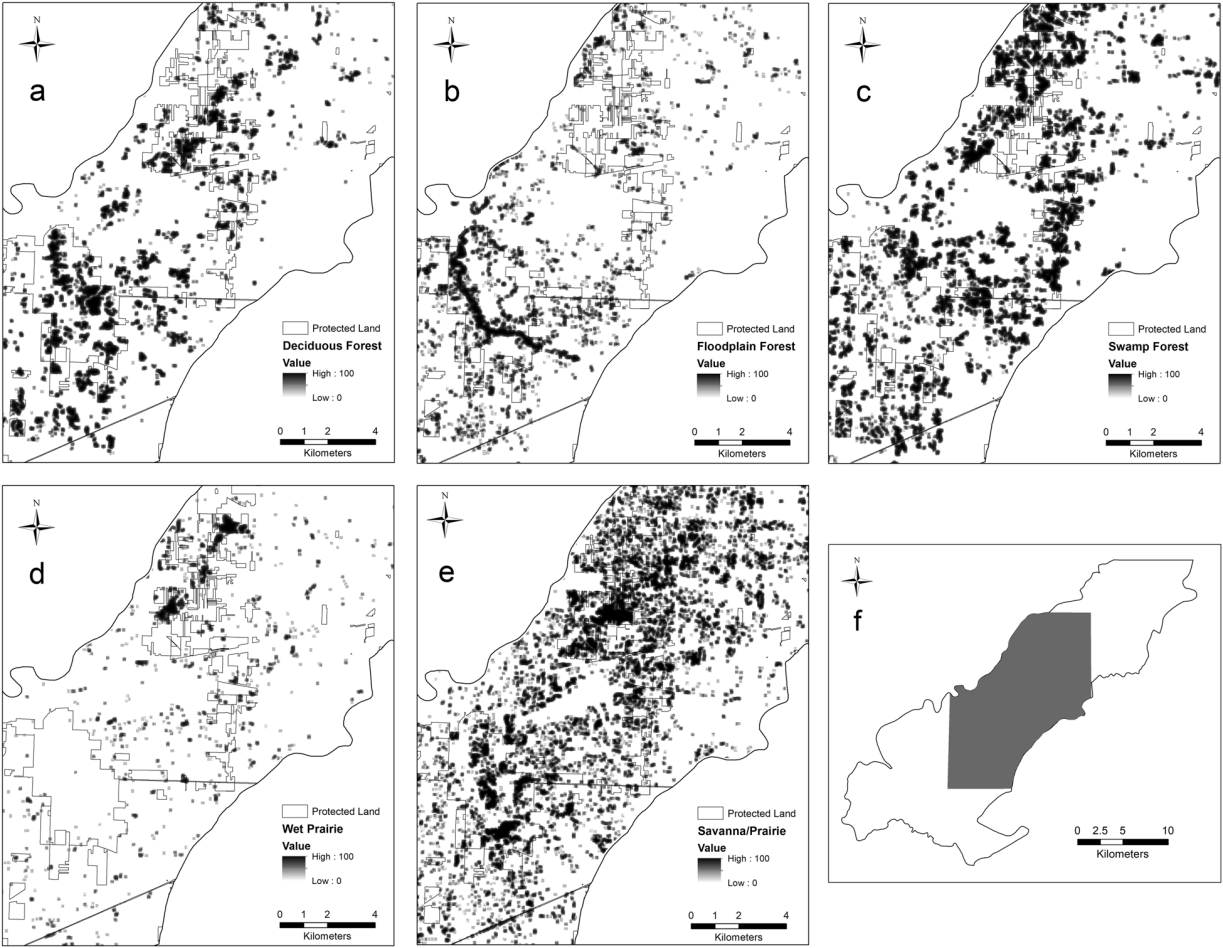
An estimate of connectivity using COHESION for the five major communities of concern (**a**–**e**) in the Ohio portion of Oak Openings (**f**); approaching 0 (lighter color) represents a physically disconnected focal class and approaching 100 (darker color) represents a cohesive aggregated focal class

**Fig. 4:**
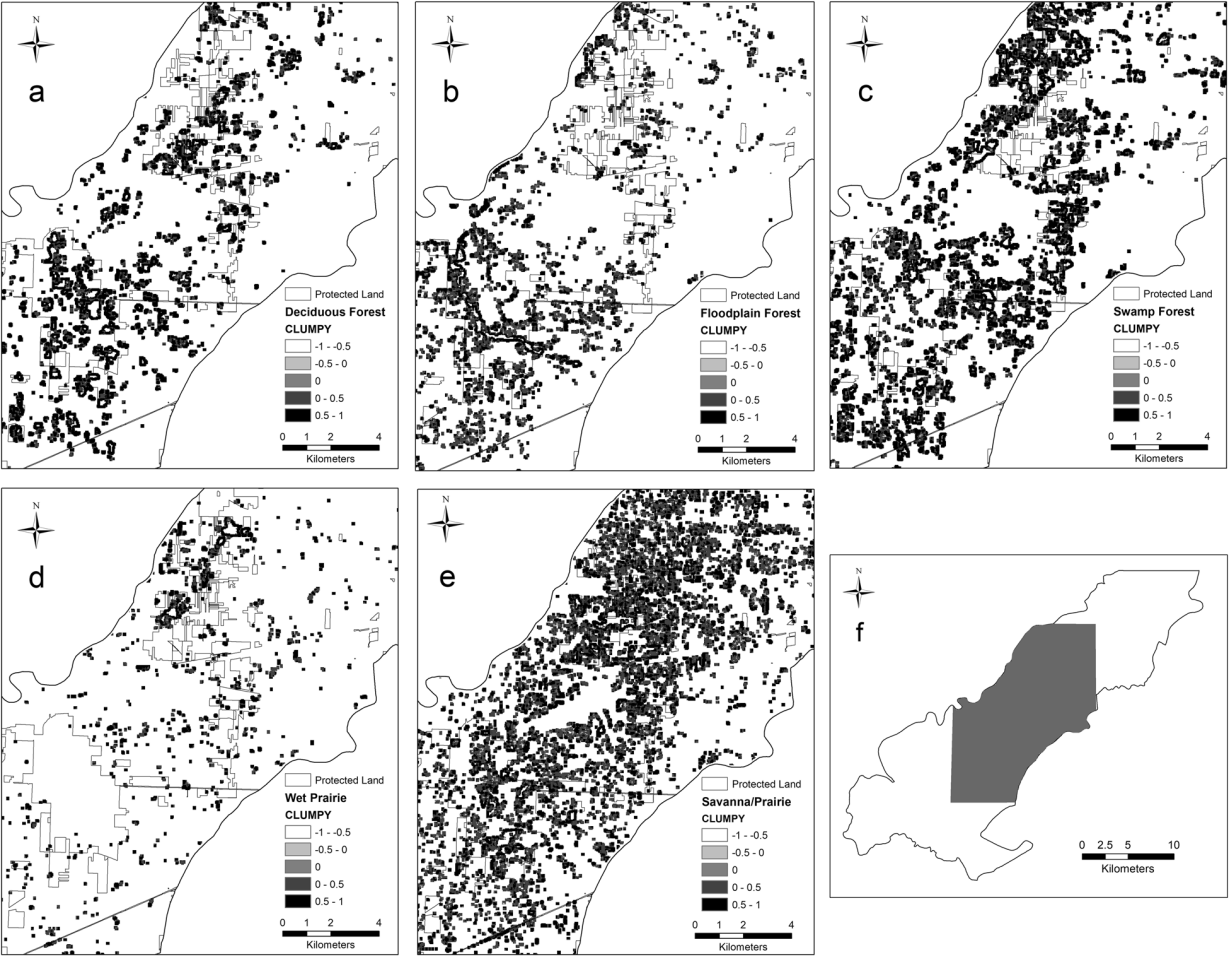
An estimate of the dispersion using CLUMPY for the five major communities of concern (**a**–**e**) in the Ohio portion of Oak Openings (**f**); approaching −1 (lighter color) represents maximally disaggregated patch type; zero represents a random distribution and approaching 1 (darker color) represents maximally aggregated patch type within a neighborhood of 120 × 120 m. Protected lands are outlined in gray

**Fig. 5:**
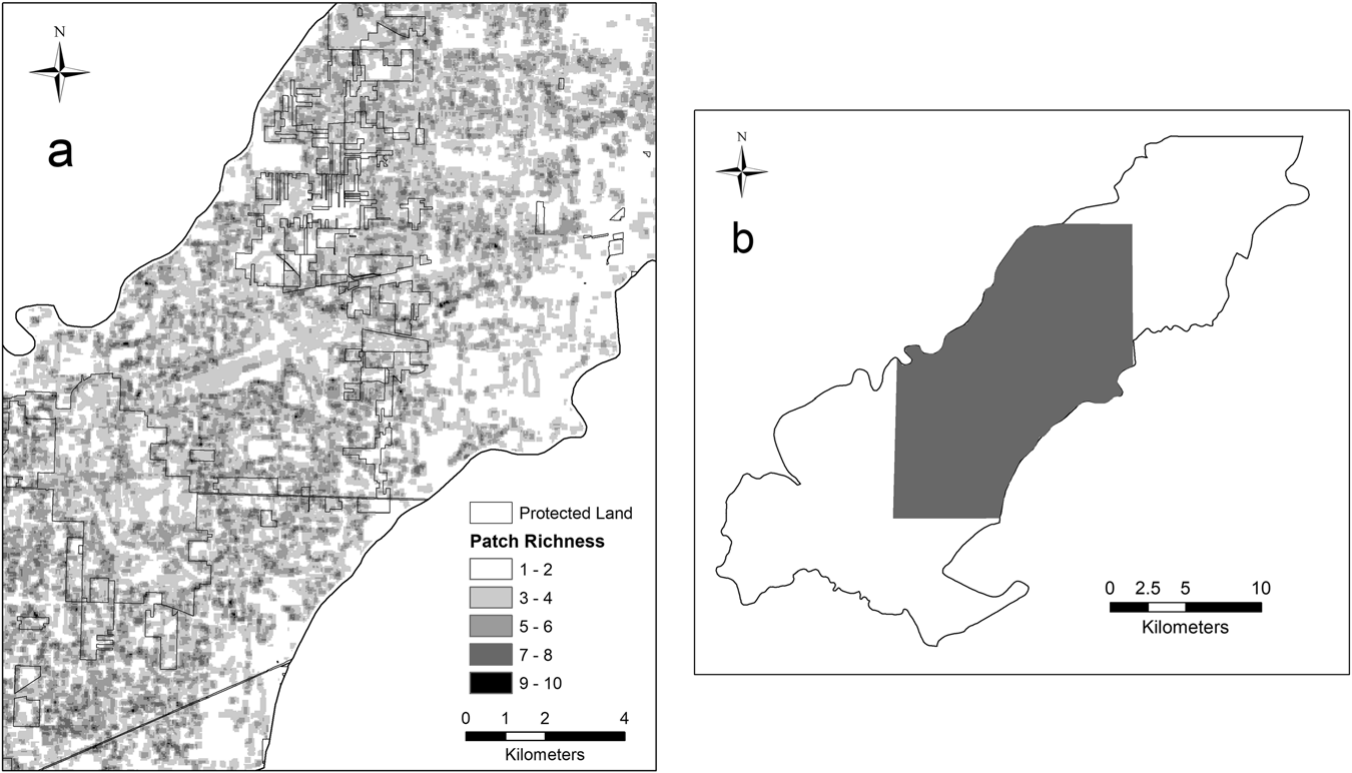
An estimate of the heterogeneity using patch richness (**a**) in the Ohio portion of Oak Openings (**b**); it shows the number of different land cover types (out of 15) that are within a neighborhood of 120 m × 120 m. Values of 1 represent a neighborhood of all the same type of land cover, whereas 10 represent the same size neighborhood with 10 different land cover types

For the five major communities of concern, we found that both floodplain and deciduous forest decreased in percent area, while swamp forest, savanna/prairie, and wet prairie increased in percent area (Fig. 2). Both floodplain forest and wet prairie decreased in average patch area, while upland savanna/prairie, swamp, and deciduous forest increased. Upland savanna/prairie increased more than 3000 patches and a similar size decreased in number of patches for floodplain forest (Table 2). Overall, the largest patch size increased for all five major communities, except upland deciduous forest. We assessed connectivity (Fig. 3), dispersion (Fig. 4) and heterogeneity (Fig. 5) for each of the five major communities of concern and overall landscape. These trends are illustrated for an example area near the Toledo airport where there are a few protected areas managed by The Metroparks of the Toledo Area and The Nature Conservancy. Cohesion and clumpiness for each of the five major communities of concern varied; however upland savanna/prairie had greater connectedness/aggregation and wet prairie had greater division/disaggregation across the area. We found that the number of patches within a 120 m × 120 m neighborhood window varied from 1 to 10 land cover classes, where ten represented greater heterogeneity.

### Map Accuracy Assessment

We evaluated 500 field assessable points with ground-truthing and orthophotos to validate the updated map. Our results were compiled in an error matrix (Table 3) and highlighted an overall accuracy of 71.2% with a kappa (measure of agreement due to chance) of 0.68. Producer’s accuracy ranged from 25% for turf/pasture to 96.4% for cropland. User’s accuracy ranged from 0% for Eurasian meadow to 100% for perennial pond. We examined the seven-level classes and found that overall map accuracy improved to 80.8% with a kappa of 0.76. Producer’s accuracy ranged from 70% for savanna to 93.2% for vacant. User’s accuracy ranged from 51.2% for savanna to 100% for perennial pond.

**Table 3:** Error matrix and accuracy for the 15-class oak openings region land cover map

	Actual land cover (reference sites)																Row total	User’s accuracy (%)
	Class	SF	FF	UD	UC	US	WS	WP	UP	SB	EM	PP	DU	RM	TP	CR		
Classified land cover (from map)	SF	*25*	1	4	0	0	0	0	0	0	0	0	0	0	0	0	30	83.3
	FF	0	*11*	8	0	1	0	0	1	0	0	0	0	0	0	2	23	47.8
	UD	4	0	*46*	0	1	0	0	0	0	0	0	0	0	0	0	51	90.2
	UC	0	0	2	*38*	0	0	0	0	0	0	0	0	0	0	0	40	95
	US	0	0	19	0	*21*	0	1	0	0	0	0	0	0	0	0	41	51.2
	WS	0	0	0	0	0	*0*	0	0	0	0	0	0	0	0	0	0	n/a
	WP	0	0	0	0	0	0	*4*	0	0	0	3	2	0	0	0	9	44.4
	UP	0	0	6	3	5	0	5	*26*	1	0	4	0	1	1	0	52	50
	SB	0	0	1	1	2	0	0	11	*5*	0	0	0	2	0	0	22	22.7
	EM	0	0	0	0	0	0	0	2	0	*0*	0	4	3	0	0	9	0
	PP	0	0	0	0	0	0	0	0	0	0	*41*	0	0	0	0	41	100
	DU	0	0	0	0	0	0	0	0	1	0	4	*37*	0	0	0	42	88.1
	RM	0	0	1	12	0	0	0	3	0	0	3	9	*48*	1	0	77	62.3
	TP	0	0	0	0	0	0	0	0	3	0	0	0	0	*1*	0	4	25
	CR	0	0	0	0	0	0	0	0	0	0	0	4	1	1	*53*	59	89.8
Column total		29	12	87	54	30	0	10	43	10	0	55	56	55	4	55	*500*	
Producer’s accuracy (%)		86.2	91.7	52.9	70.4	70.0	n/a	40.0	60.5	50.0	n/a	74.5	66.1	87.3	25.0	96.4		
	*Kappa* = *0.68*																	
Key	*SF* Swamp Forest					*US* Upland Savanna					*SB* Sand Barrens					*RM* Residential/Mixed		
	*FF* Floodplain Forest					*WS* Wet Shrubland					*EM* Eurasian Meadows					*TP* Turf/Pasture		
	*UD* Upland Deciduous Forest					*WP* Wet Prairie					*PP* Perennial Ponds					*CR* Cropland		
	*UC* Upland Coniferous Forest					*UP* Upland Prairie					*DU* Dense Urban							

### Protected Areas and Restoration Efforts

**Fig. 6:**
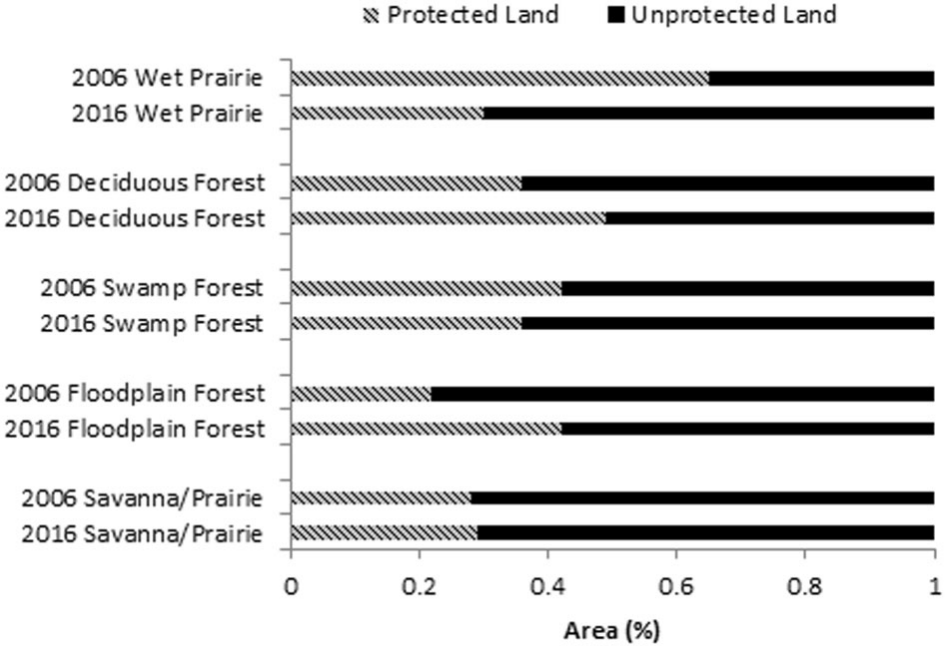
Summary of percent area for each of the major communities of concern for both protected (pattern) and unprotected (no pattern) lands in the Ohio portion of Oak Openings Region for the original (Schetter and Root 2011) and updated land cover map

We examined the total of permanently protected areas within Oak Openings Region of Northwest Ohio and found that 12% of the land is currently in protection with 10% of natural/seminatural land cover in protection. Our analysis focused on changes over time for the five major communities of concern within protected areas and found that upland savanna/prairie increased by 1%, upland deciduous forest increased by 13%, and floodplain forest increased by 20%, while wet prairie decreased by 35% and swamp forest decreased by 6% over time (Fig. 6). Positive changes within protected areas corresponded with negative changes within unprotected areas; for example, a 20% increase in protected floodplain forest corresponded with a 20% decrease in unprotected areas. The patch characteristics within protected areas varied for the five major communities of concern (Table 4). Upland savanna/prairie tripled in the number of patches with the average patch area and largest patch size, respectively, increasing by 0.17 ha and 0.4% over time. We found that upland deciduous forest decreased over time, except in average patch area. Upland deciduous forest lost 221 patches and the largest patch size dropped by 0.55%.

**Table 4:** Summary of land cover results for all currently protected parks and preserves in Oak Openings Region for the original (Schetter and Root 2011) and updated land cover maps

	# patches		LPI (%)		ED (m/ha)		AVG area (ha)		Cohesion	
Land cover type	2006	2016	2006	2016	2006	2016	2006	2016	2006	2016
Savanna/Prairie	452	*1515*	0.97	*1.37*	30.24	*99.21*	0.6	*0.77*	82.98	*86.98*
Floodplain Forest	1465	923	3.98	3.7	105.06	72.29	0.62	*0.68*	88.86	*90.88*
Swamp Forest	1170	744	0.85	*1.09*	83.36	16.11	0.52	*1.53*	81.59	*88.87*
Deciduous Forest	699	478	1.6	1.05	88.18	57.18	1.53	*1.95*	90.51	89.8
Wet Prairie	27	*361*	0.2	*0.73*	1.96	*99.21*	0.94	0.57	81.56	*84.24*

Italic numbers represent increases over time

*AVG* average, *NP* number of patches, *LPI* largest patch index, *ED* edge density, *COHESION* patch cohesion index

We examined land cover changes within Oak Openings Preserve and found that both upland deciduous and coniferous forest decreased in total area over time, respectively by 64 ha and 161 ha, while floodplain and swamp forest increased in total area over time, respectively by 17 ha and 23 ha. All forest land covers lost a range of 32–176 patches; swamp forest had the largest loss and conifer forest had the smallest loss of patches. As expected from targeted restoration efforts towards dry early successional land cover, we found an increase in total area of 175 ha for upland prairie, 66 ha for upland savanna, and 53 ha for sand barren. Dry early successional land cover all gained a range of 61–216 patches, with upland prairie had the greatest gain and upland savanna had the smallest gain of patches.

## Discussion

### Land Cover Maps can Reveal Spatial and Temporal Changes

**Fig. 7:**
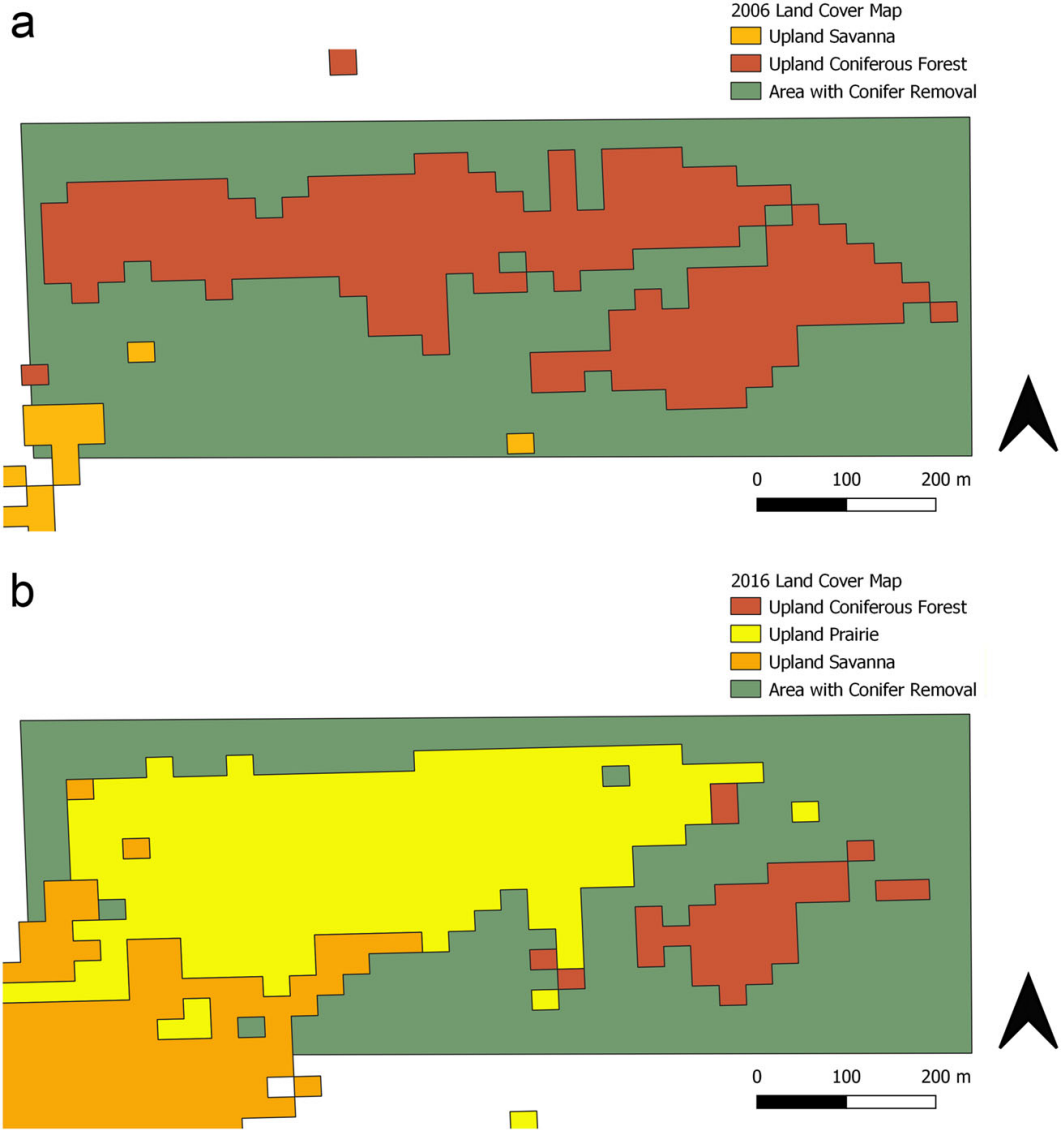
Enlarged area of the original 2006 (**a**) (Schetter and Root 2011) and updated 2016 (**b**) land cover map within Oak Openings Preserve where pine removal occurred by land managers in 2010

Significant changes often occur over time and it is critical to understand these changes when prioritizing land management and conservation in a region (Fardila et al. 2017). Updating the land cover map provided a useful approach to examine larger-scale trends across the region and should be done periodically (e.g., every 10 years). In the original land cover map of Oak Openings Region, much of the land had been converted to human-modified land cover classes (73%), while <3% was covered by upland early successional habitats, such as prairie and savanna (Schetter et al. 2013). We predicted that natural areas, especially for targeted communities, would have increased over time because of restoration and land acquisition efforts. Our new land cover classification has shown that even though human-modified land cover classes have increased to 76.6%, early successional habitats have also increased to 7%. This increase in early successional habitats (e.g., increasing wet prairie (0.1 to 0.3%) and upland prairie (1.3 to 3.6%) is likely a result of the work of the GRI to increase the amount of protected lands and promote ecological conservation through restoration and enhancement of critical natural areas (Abella et al. 2007). For example, the conifer management plan focused on improving timber stands and completely removing conifers in specific locations to convert to native oak woodlands, savannas, and prairies (Metroparks Toledo 2020). This has been effective for benefitting native species and restoring native communities for at least 14 years with a focus on converting pine plantations into savannas and prairies (Abella et al. 2017). Since 2002, 140.8 ha of pine remain after the 2018–2019 management plan and 187.8 ha successfully restored to prairie and savanna (Schetter and Gallaher 2019). The desire to restore open-structure habitat is likely why we found a larger increase in savanna/prairie, while we found a decrease in forest land covers. However, not all changes may be from human modifications, such that natural recovery or disturbances also play a role in restructuring land cover. For example, land managers had a rare opportunity to examine how a natural disturbance can influence restoration efforts from a tornado that hit a 23-year old restoration site in 2010 (Abella et al. 2018). Although the restoration is occurring at specific local sites, the changes detectable at the regional scale provide a view of the larger impacts the collective efforts have had on the overall landscape (Fig. 7).

The efforts to protect and restore early successional habitat have been successful, as demonstrated by an increase in habitat cover by 11%; however, other changes were not as favorable. The updated land cover map also revealed declines in vacant (–7.6%) and forest and woodland (−5.4%) land cover. The loss of forest is not surprising given factors such as the rise of invasive species (e.g., Emerald Ash Borer, Gypsy Moth, *Lymantria dispar*, Linnaeus), disease (e.g., oak wilt, *Ceratocystis fagacearum*) and continuing development and fragmentation in the region. Deforestation is already widespread from direct loss and expanding anthropogenic land cover; however, remnant forests are additionally suffering from changes in structural characteristics such as more isolation, smaller size, and greater area of edge habitat (Haddad et al. 2015). Additional structural changes occur from both of the invasive species that have led to large mortality rates and defoliation of both ash (*Fraxinus spp*.) and oak (*Quercus spp*.) trees, resulting in a loss of area and connectivity among forest habitat (Knight et al. 2013; Domínguez-Begines et al. 2018). Although some of these species invaded Northwest Ohio before our original land cover map, we are now detecting the long-term consequences. Invasive species can have a secondary release into the surrounding natural landscapes from their established focal sources in urban areas (Alston and Richardson 2006; von der Lippe and Kowarik 2008). Therefore, the growing development and fragmentation in this region may increase the susceptibility of this biodiverse region to invasive species and other threats.

### Improvements in the Land Cover Mapping

Oak Openings Region is a biodiversity hotspot characterized by its highly heterogeneous landscape, making it an excellent model to assess mapping accuracy. Overall, our map accuracy was 73.8%, which was an improvement over the 60% accuracy of the original land cover map. The improved accuracy is likely a result of the additional spectral data available in Landsat 8 images versus Landsat 5 (Poursanidis et al. 2015). However, we verified only a selection of the land cover classes as a result of time constraints. These selected land covers may be easier to detect, such as perennial pond and upland coniferous forest, and may not represent the accuracy for all land cover classes. Some caution is warranted, therefore, in the absence of further verification. For some classes, such as upland conifer, we were highly successful in accurately matching the satellite image to the real world. Whereas our map poorly predicts wet prairie based on the ground truth points, which were primarily upland prairie. We found a decrease of 35% over time for wet prairie within protected lands, despite corroborated wet prairie restoration which occurred over the last decade. This could be a result of overclassifying wet prairie across unprotected lands and we suggest increasing training data for this land cover class across the region. The original training data provided a reasonable classification, but some classes were too broad and overlapped with other land cover classes. With additional training points, the classification can be improved providing a more accurate map. In addition, technological advancements may increase accurate mapping with the release of newer Landsat data within the next 10 years. Our recommendation would be to identify additional training points within land cover classes of concern based on conservation and management goals for the region of interest.

### Complementary Approaches can Increase Understanding of Changes

**Fig. 8:**
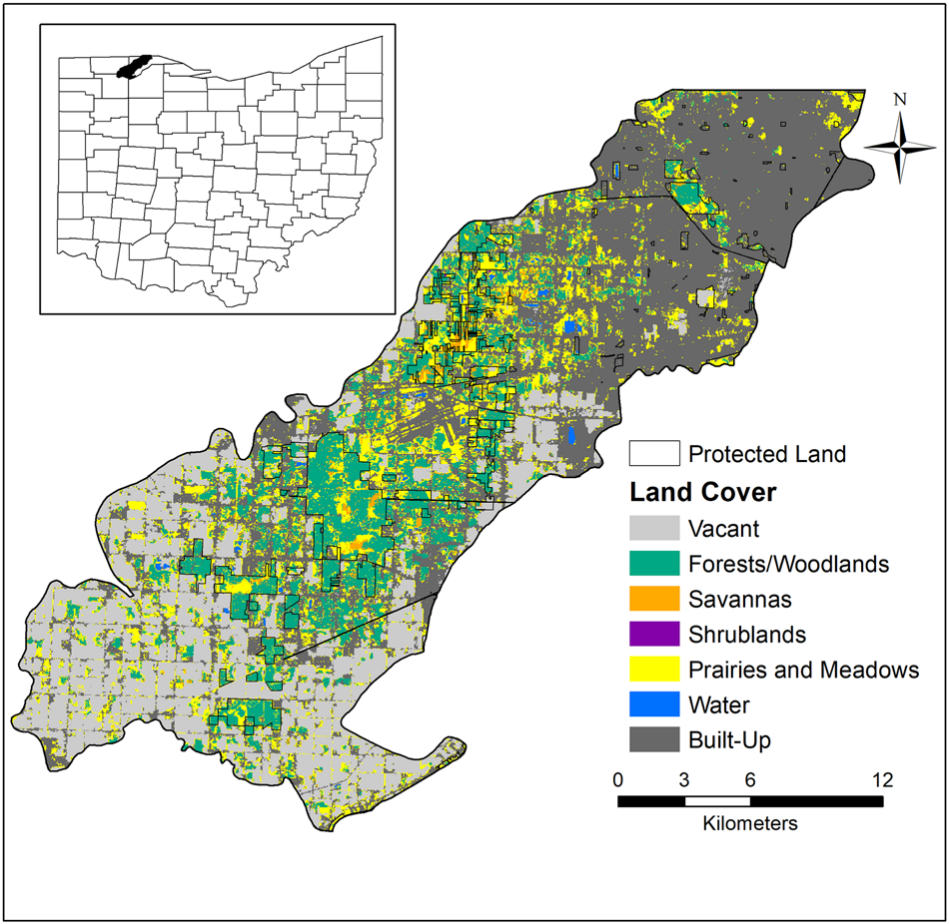
Simplified 7-class land cover map for the Northwest Ohio portion of Oak Openings Region. Protected land outlined in black

One unique aspect of our study is the incorporation of changes in landscape configuration as a measure of functionality, which is often underutilized when examining restoration efforts. Therefore, in addition to changes in the amount of habitat, how it is distributed has also changed. These changes are readily visible when we simplified the land cover map to highlight the seven-level class map, as seen in Fig. 8. The results of our fragmentation analysis suggest that the number of patches is increasing, particularly for specific land cover classes (e.g., upland prairie, wet prairie). While the overall proportion of some of these land cover classes has increased, the distribution is one of small isolated patches, which are likely to be far more vulnerable to further degradation and invasion. Although habitat configuration is more important when levels are below 10–30%, it is important to incorporate these changes within management plans and create threshold tests for ecological processes and species (Homan et al. 2004). Like our study, others have found increased fragmentation (Hanberry and Abrams 2018) and a complex pattern of fragmentation (Fardila et al. 2017) across the landscape over time. Conservation targets that examine the pattern, as well as the amount of habitat, can help counter the piecemeal restoration that typically occurs in human-modified landscapes, therefore managing the entire mosaic rather than individual pieces (Lindenmayer et al. 2008). With many of the protected areas in this region surrounded by anthropogenic features, it would be beneficial to focus on increasing patch size to buffer the impacts of anthropogenic factors. The surrounding matrix often has large influences on populations and communities often much more than processes within remnant focal patches (Wiens 1995), and many species responses are often scale-dependent (Turner et al. 2001). This approach provides a way to prioritize connectivity efforts to offset increasing fragmentation and fosters planning at larger scales, which has been recommended by others (e.g., Fardila et al. 2017; Watson et al. 2017).

While the land cover and fragmentation analysis can reveal the changes within the landscape, it is limited to vegetation or structural characteristics. The goal for conservation is healthy functional ecosystems, which requires an assessment of quality as well as quantity. We can address this need by combining land cover data with target indicator species to identify habitat quality rather than just habitat quantity. For example, species distribution models were developed for the red-backed salamander (*Plethodon cinereus*, Green) using occurrence data, land cover, and soil maps. Red-backed salamanders are widely distributed among upland deciduous forest with population densities reaching up to 0.9–2.2 individuals/m^2^ (Pough et al. 1987). They are found preferably under deciduous leaf litter over coniferous forest (Renaldo et al. 2011) and represent an excellent model for examining habitat functionality. The resulting model highlights functional upland deciduous forest from the perspective of a native occupant and identifies important habitat in terms of both area and function. This type of species distribution model presents other landscape features that provide additional support for native species, such as floodplain forest and specific soil types. Complementary approaches such as occupancy modeling in conjunction with land cover maps aid in conservation planning of both quality and quantity of functional native ecosystems.

## Conclusions

The study explored the effects of the ground management on LULC over a 10-year period in Oak Openings Region. We found positive changes for the two early successional communities which were key land management targets. This suggests that local scale (i.e., on the ground changes) from local land managers can be visible from landscape viewpoints (i.e., satellite data) over time. Land managers would benefit from examining regional maps for overall long-term conservation plans to preserve a variety of natural habitats to maintain the natural biodiversity and ecosystem function. Not only is this approach valuable to evaluate the larger scale impacts of small-scale restoration projects, but it also highlights critical features (e.g., connectivity) that can identify priorities for future acquisition and conservation and targets for management activities. Our results illustrate the challenges that land managers face, in that focused restoration can help some facets of the landscape, but degradation and fragmentation are likely to continue as human-modified land covers expand and/or encroach on natural habitats. Project-by-project approach may not be as effective in mitigating these negative effects, therefore there is a need to have a comprehensive large-scale view of conservation planning to effectively mitigate future changes.

## Supplementary information

Supplementary Information
