# Supplementary material for: Examining Land Use Changes to Evaluate the Effects of Land Management in a Complex, Dynamic Landscape
Source: Environ Manage. 2020 Jun 22;66(3):333–47. doi: 10.1007/s00267-020-01316-2 (PMC7434799; doi:10.1007/s00267-020-01316-2)
Supplement: Supplementary file 1 — Supplementary Information [file 267_2020_1316_MOESM1_ESM.pdf]

Examining Land Use Changes to Evaluate the Effects of Land Management in a Complex, Dynamic Landscape
Environmental Management

Amanda K. Martin[1,2] and Karen V. Root[1]

[1]Department of Biological Sciences, Bowling Green State University, Bowling Green, OH 43403 USA
[2]Corresponding Author: Amanda K. Martin, amandkm@bgsu.edu

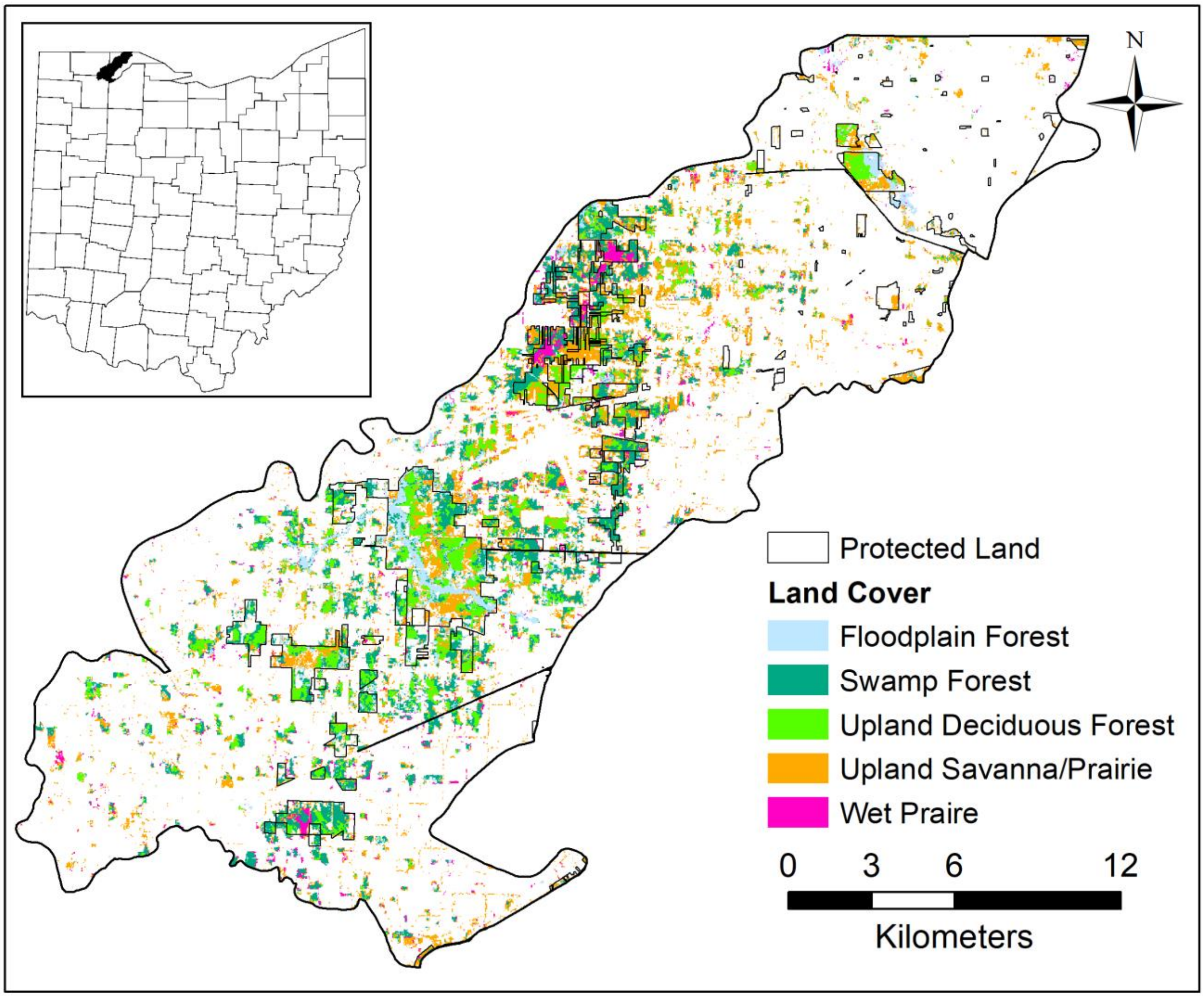

Supplement Fig. 1 Map of the five major communities of concern for Oak Openings Region in Northwest Ohio with protected lands outlined in black

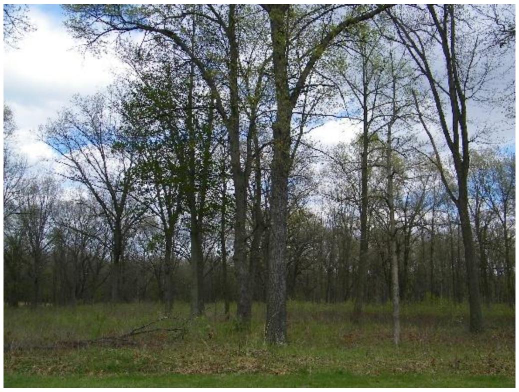

(a) upland savanna

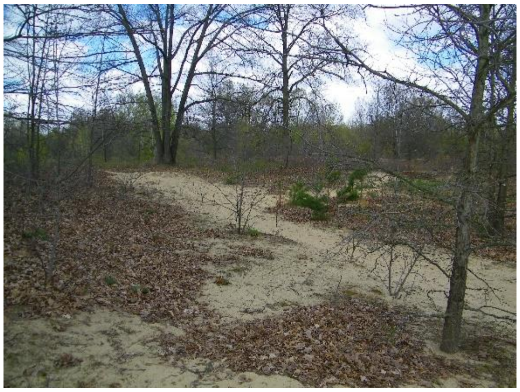

(b) sand barren

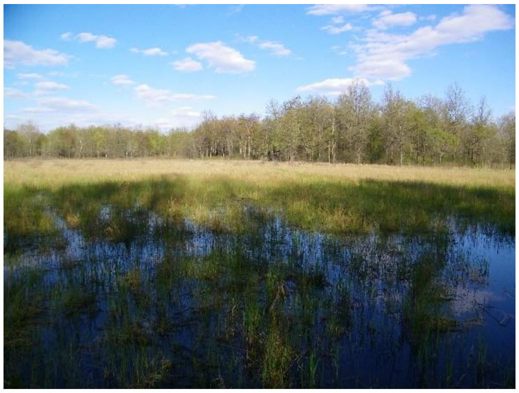

(c) wet prairie

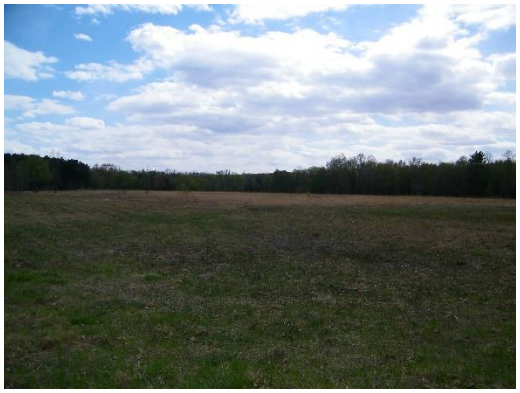

(d) upland prairie

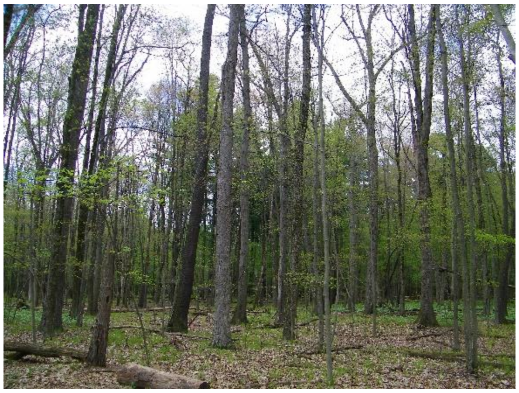

(e) upland deciduous forest

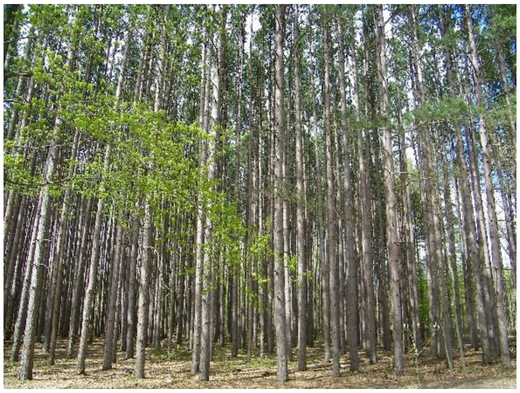

(f) upland coniferous forest

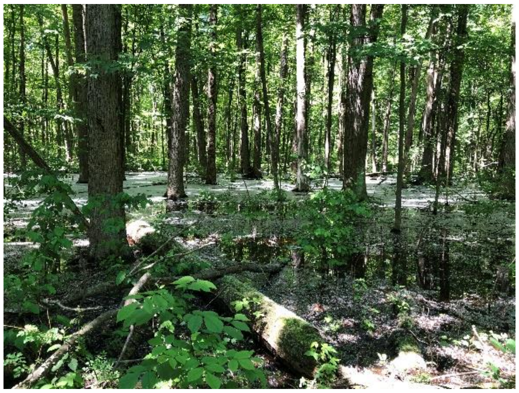

(g) swamp forest

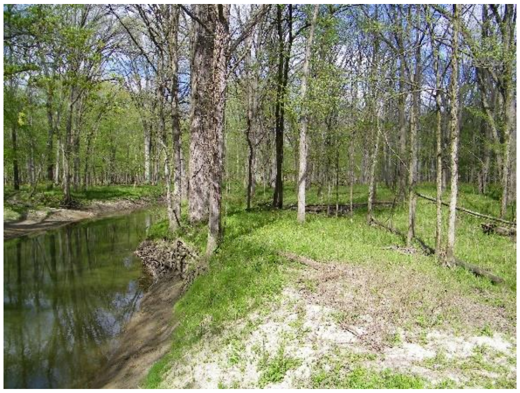

(h) floodplain forest

Supplement Fig. 2 Representative photographs for eight major land cover classes (a-h)
